# Melanogrit potentiates melanogenesis by escalating cellular tyrosinase activity and MITF levels via pERK inhibition

**DOI:** 10.1042/BSR20231324

**Published:** 2024-01-09

**Authors:** Acharya Balkrishna, Savita Lochab, Sudeep Verma, Jyotish Srivastava, Rishabh Dev, Anurag Varshney

**Affiliations:** 1 Drug Discovery and Development Division, Patanjali Research Foundation, NH-58, Haridwar 249405, Uttarakhand, India; 2 Department of Allied and Applied Sciences, University of Patanjali, Patanjali Yog Peeth, Roorkee-Haridwar Road, Haridwar 249405, Uttarakhand, India; 3 Patanjali Yog Peeth (UK) Trust, 40 Lambhill Street, Kinning Park, Glasgow G41 1AU, U.K.; 4 Vedic Acharya Samaj Foundation, Inc. NFP, 21725 CR 33, Groveland, FL 34736, U.S.A.; 5 Special Centre for Systems Medicine, Jawaharlal Nehru University, New Delhi, India

**Keywords:** B16F10, cellular tyrosinase activity, keratinocytes-melanocyte co-culture, Melanogenesis, MITF

## Abstract

Vitiligo is characterized by the development of white patches on the skin either due to the loss of functional melanocytes or perturbations in the melanogenesis pathway. In the present study, we investigated the therapeutic potential of herbo-mineral formulation, Melanogrit in neutralizing the white patches in the skin. The study utilized UPLC/MS-QToF technique to determine the diversified phytochemical profile in Melanogrit. The murine B16F10 cells when treated with Melanogrit underwent morphological changes, including increased angularity, enlarged cell size, and greater dendritic protrusions. To establish an equivalent model to study melanogenesis, we carefully optimized the dosage of α-melanocyte stimulating hormone (αMSH) in B16F10 cells as an alternative to using melanocyte-keratinocyte cocultures. The study determined a sub-optimal dose of αMSH (0.2 nM) in B16F10 cells that does not manifest any measurable effects on melanogenesis. In contrast, Melanogrit when used in conjunction with 0.2 nM αMSH, induced a dose-dependent increase in extracellular and intracellular melanin levels. Melanogrit transcriptionally up-regulated the decisive genes of the melanogenesis pathway, MITF, TYR, and TRP1, which was evident from the increased cellular tyrosine activity. Our findings also demonstrated that Melanogrit ameliorated the MITF protein levels by inhibiting pERK; notably without involving GSK3β in the process. Taken together, our findings strongly suggest that Melanogrit has the potential to stimulate melanogenesis, making it a promising candidate for clinical applications in the treatment of white skin patches that develop in vitiligo patients.

## Introduction

Vitiligo is a chronic skin depigmentation condition that affects approximately 1–2% of the global population [1]. It is characterized by the appearance of white macules and patches anywhere on the skin including the exposed skin of the face, hands, and legs [2]. Although skin depigmentation may not be fatal, but it is debilitating and has a negative impact on a person's psychosocial behaviour, which has been linked to social stigma, depression, and anxiety [3–5]. The precise aetiology of onset and progression of vitiligo is insufficiently understood, primarily due to its multifaceted pathogenesis engaging autoimmunity, melanocyte destruction, extracellular matrix, intracellular redox conditions and signaling cascades [6]. However, all these factors ultimately result in the complete or partial loss of functional melanocytes in the skin.

Melanocytes hold specialized organelles called melanosomes that synthesize the skin pigment, melanin through the process known as melanogenesis. Sequential enzymatic reactions within the melanocytes convert the amino acid, tyrosine into either form of melanin, eumelanin, and pheomelanin. Keratinocytes residing in the skin epidermis release various paracrine signals, like, α-melanocyte stimulating hormone (αMSH), adrenocorticotropic hormones (ACTH), stem cell factor (SCF), and endothelin-1 (ET-1) that trigger melanogenesis in the neighbouring melanocytes. The αMSH, being the major signal, binds to the receptor, MC1R on melanocytes, which in turn activates the cAMP/CREB key signaling cascade to trigger the transcriptional activation of key genes of the melanogenesis pathway, including MITF (microphthalmia-associated transcription factor), tyrosinase (TYR), tyrosinase-related protein 1 (TRP1), dopachrome tautomerase (DCT), and PMEL [7]. Melanin, thus produced inside melanocytes is subsequently transferred to keratinocytes through melanosomes, imparting both colour and protection from the damaging effects of sunlight.

Mainstream treatment modalities for vitiligo include phototherapies using ultraviolet A (UVA), narrow band ultraviolet B (NB UVB), often in combination with topical or systemic administration of steroids, calcineurin inhibitors, vitamins, analogues of psoralen. While these treatments demonstrate efficacy in managing symptoms, they can sometimes lead to side effects cytotoxicity, phototoxicity, blistering, and even flare-up into skin cancer over long-term usage [8,9]. In this line, natural compounds, herbs, and their extracts may provide a safe and effective treatment for white skin patches in vitiligo, either by shielding melanocyte destruction due to oxidative stress or by inducing repigmentation in melanocytes.

Indian ancient medicinal system, Ayurveda has mentioned the herbs and herbal–mineral combinations that could induce pigmentation in the white patches. These herbal combinations modulate signal transduction pathways that could reactivate melanin biosynthesis or increase the arborization of melanocytes to reach distantly located keratinocytes. On the similar lines, our study aims to explore the herbal formulation, namely, Melanogrit for the mechanism of action. Melanogrit is a trademarked herbal formulation manufactured and marketed by Divya Pharmacy, Haridwar, India. It has been approved for clinical use by the respective regulatory agencies (vide manufacturing license number Uttra.Ayu-67/2005). In addition, observational clinical data from the empirical studies, involving 151 participants, has demonstrated treatment benefits in individuals with vitiligo or leukoderma (Data not shown, to be published elsewhere).

Our study here aims to elucidate the underlying mechanism of the therapeutic potential of Melanogrit against vitiligo. We show that Melanogrit could potentiate melanogenesis in murine melanocytes, B16F10 cells. Further, experiments were designed employing B16F10 cells alone or co-cultured with keratinocytes to decipher the mechanism of melanogenesis induction through Melanogrit. We indeed determined that B16F10 cells spiked with a sub-optimal dose of αMSH (0.2 nM) was the apt model to study melanogenesis, here. The 0.2 nM dose of αMSH had no effect on the transcriptional and translational levels of the melanogenesis pathways. The same was supported by the phenotypic observations including dendrite formations and melanin biosynthesis. Melanogrit, enhanced the transcriptional levels of MITF, TYR, and TRP1, the key players of melanogenesis in B16F10 cells that were sub-optimally stimulated with αMSH. Melanogrit treatment potentiated the cellular tyrosinase activity and melanin levels, both, extracellularly and intracellularly. The kinases, ERK and GSK3β, have already been reported to regulate the ubiquitin-proteasomal degradation of MITF; Melanogrit, significantly reduced the pERK levels while increasing the protein levels of MITF. Interestingly we observed almost constant levels of pGSK3β indicating the mechanism of Melanogrit in modulating MITF levels is independent of GSK3β. Based on the above findings, we propose Melanogrit as an effective therapy for vitiligo.

## Material and method

### Test article procurement

The test article, Melanogrit (Internal Code # CHIH/MEAA/0322/2416) was sourced from Divya Pharmacy, Haridwar, India.

### Chemicals

Cell culture media, Phenol Red Free DMEM (Dulbecco’s Modified Eagle Medium), Antibiotic-Antimycotic (100×), and Dulbecco’s Phosphate Buffered Saline were purchased from GIBCO, U.S.A. Fetal Bovine Serum (FBS) and 0.5% Trypsin-EDTA was purchased from Hi-media Laboratories Pvt Ltd, India. The reagents, dimethyl sulphoxide (DMSO), 3,4-dihydroxy-L-phenylalanine (L-DOPA), α-melanin stimulating hormone (αMSH), Melanin were procured from Sigma, U.S.A. General laboratory chemicals, sodium hydroxide, disodium hydrogen phosphate, sodium dihydrogen phosphate, and Tween-20 were obtained from Merck India Pvt. Ltd. The reagents, alamar blue and bovine serum albumin fraction V, were purchased from Hi-media Laboratories Pvt Ltd, India.

LCMS grade solvents methanol, acetonitrile, and formic acid were obtained from Honeywell, Germany. HPLC grade acetonitrile was procured from Finar (Gujarat, India) while methanol and acetic acid were obtained from Rankem (Maharashtra, India). Deionized water was obtained from a Milli Q system (Millipore, Billerica, MA, U.S.A.). The standards for UHPLC, protocatechuic acid (Potency: 99.5%), and methyl gallate (Potency: 97.3%) were procured from Natural remedies, India, while the commercial source for vanillic acid (Potency: 98.20%) and psoralene (Potency: 98.7%) was purachased from Sigma Aldrich, U.S.A. Isopsoralene (Potency: 97.01%) on the other hand was inhouse isolated from a herbal source and internally validated.

### Cell line maintenance

The murine melanocytes, B16F10 cells were procured from ATCC licensed cell line repository located at National Centre for Cell Sciences (NCCS, Pune, India). Human keratinocytes and HaCaT cells were procured from Krishgen Biosystems, India (Cat # KCC0090). The cell lines were routinely propagated in phenol-red-free DMEM (Dulbecco’s Modified Eagle Medium) supplemented with 10% Fetal Bovine Serum (FBS) and 1% Anti-Anti (Antibiotic-Antimycotic) under the growing conditions maintained at 37°C and 5% CO_2_ in a humified incubator. Cryopreserved cells were passaged at least twice before using for the experiments in this study.

### Co-culture experiments

HaCaT cells were seeded in a six-well tissue culture plate at a density of 1.0 × 10^6^ cells per well. After 24 h, B16F10 cells were seeded in the well already seeded with HaCaT cells. The seeding density of B16F10 cells was one-fifth of the HaCaT cells, i.e. 0.2 × 10^6^ cells/well. Twelve hours after seeding B16F10 cells, the co-culture setup was treated with indicated treatments of test articles.

### Alamar blue cell viability assay

Cells at a density of 1.0 × 10^5^/ml were seeded in 96-well tissue culture plates. After 24 h, Melanogrit prepared in cell culture media was used to treat cells at concentrations with a half-log fold difference, ranging from 1 to 300 μg/ml for 72 h. Three hours before the indicated time period of 72 h, Alamar blue at a working concentration of 15 μg/ml was added to the respective media. The fluorescence was recorded in Envision multi-plate reader (PerkinElmer, Waltham, MA, U.S.A.) at excitation and emission wavelength of 560 and 590 nm, respectively. Percent cell viability was calculated using the following formula, wherein AU is arbitrary fluorescent units:

$$\%\;\text{Cell Viability}\;=\left[\frac{\text{(AU Treated}-\;\text{Blank)}}{\left(\text{AU Untreated}-\;\text{Blank}\right)}\right]\times 100$$

### Intracellular and extracellular melanin determination

Melanin determination was performed as described previously [10]. B16F10 cells treated with indicated Melanogrit concentrations for 5 days. The cell media was collected and imaged with a camera for visualizing colour change in the media. Melanin content in the extracellular media was measured by recording the absorbance at 405 nm and further quantified against the melanin standard.

For intracellular melanin evaluation, once the extracellular media was removed, cells were washed with PBS, and collected in the form of pellet in microcentrifuge tubes. The color change from light brown to black was observed and subsequently captured by the camera (Canon, Japan). The cell pellets were resuspended in 10% DMSO in 1 N NaOH and heated at 80°C for 2 h. Total intracellular melanin was then determined by recording the absorbance of the dissolved cell pellet at 405 nm. Absolute melanin content was quantified against melanin standard and further normalized by protein concentration of respective pellet determined by Bradford assay (G Bioscience, India). Absolute melanin concentration in each sample was normalized with respect to their protein concentrations. Once determined, fold change was calculated with respect to the untreated B16F10 cell pellet. The absorbance of samples was recorded in Envision multi-plate reader (PerkinElmer, Waltham, MA, U.S.A.)

### Cellular tyrosinase activity

Cellular tyrosinase activity was measured as discussed elsewhere [11]. B16F10 cells treated with Melanogrit at the indicated concentration for 72 h were washed with PBS and lysed in sodium phosphate buffer (0.1 M, pH 6.8) containing 1% Triton X-100 followed by repeated snap freeze–thaw cycles. The reaction mix for cellular tyrosinase activity contains 50 μl of 5 mM L-DOPA and 50 μl of the cell lysate that was further incubated at 37°C for 30 min. Absorbance measured at 475 nm in Envision multi-plate reader (PerkinElmer, Waltham, MA, U.S.A.) was normalized with protein concentration determined by Bradford assay (G Bioscience, India).

### L-DOPA staining

B16F10 cells post 72 h of Melanogrit treatment at different concentrations were incubated with 0.1% L-DOPA at 37°C for 6 h, as described previously [12]. Stained cells were observed and imaged under a bright-field microscope (Olympus BX43, MANTRA, Perkin Elmer, U.S.A.).

### Phalloidin staining

B16F10 cells were stained using Alexa Fluor 594 Phalloidin according to the manufacturer’s protocol. Briefly, Phalloidin B16F10 cells were grown on coverslips in six-well tissue culture-grade plates. Cell treatment with Melanogrit was performed at indicated concentrations for 72 h. Cells were washed with PBS and fixed with 3.7% formaldehyde in PBS for 15 min at room temperature. Fixed cells were permeabilized with 0.1% Triton X-100 prepared in PBS for 10 min. Cells were then incubated in 1% BSA prepared in PBST (PBS containing 0.05% Tween 20) for 1 h. Cells were incubated with Alexa Fluor 594 Phalloidin stain for 30 min at room temperature. The stain was diluted at 1× working concentration in PBST from 400 × stock concentration (∼5 mg/ml) prepared in DMSO. Cells were washed thrice with PBST and were mounted on slides using Vectashield antifade mounting medium with DAPI (H1200, Vector Labs, U.S.A.). Cells were examined and imaged with a fluorescent microscope (Olympus BX43, MANTRA, Perkin Elmer, U.S.A.). The images were further analyzed using the trace and measure plugin in Neurite tracer (ImageJ) [13].

### RNA extraction and quantitative real-time PCR

Total RNA was extracted and purified from B16F10 cells post 72 h of Melanogrit treatment using RNeasy mini kit (Qiagen, Germany) according to the manufacturer’s protocol. The RNA was quantified and tested for quality by determining the absorbance ratio at 260 and 280 nm in Nabi spectrophotometer. 1 μg of RNA was utilized from each sample to synthesize cDNA (Verso cDNA synthesis kit, Thermo Fisher Scientific, U.S.A.), according to the manufacturer’s protocol. A reaction of the synthesized cDNA with SYBR Green (PowerUp SYBR Green Master Mix, Thermo Fisher Scientific, US.A.). and respective forward and reverse primer sets were subjected to quantitative real-time PCR (qTOWER^3^, Analytik Jena, Germany). The thermocycling program performed amplification for 35 cycles wherein each cycle consisted of denaturation at 95°C for 30 s, annealing at 55°C for 30 s, and extension at 72°C for 20 s. Actin gene was used as a housekeeping control gene to calculate relative expression levels. The normalized expression fold change was calculated with 2^−ΔΔCT^ method [14]. The following are the sequences of the primers used for amplifying targets genes in murine B16F10 cells:

**Table:** 

MITF:	Forward 5′-GGCCAAGGCAGAGCAACTT-3′
	Reverse 5′-GCCCATGGTGGCAAGCT-3′
TYR:	Forward 5′- ATAGGTGCATTGGCTTCTGG-3′
	Reverse 5′- CCAACGATCCCATTTTTCTT -3′
TRP1:	Forward 5′- GAGTGACATCC TGTGGCTCA -3′
	Reverse 5′- CGATACCCTGGGAACACTTT -3′
β-Actin:	Forward 5′- ACGGCCAGGTCATCACTATTG -3′
	Reverse 5′- TGGATGCCACAGGATTCCA -3′

### Preparation of whole cell lysates and Western blotting

B16F10 cells were treated with Melanogrit at 1, 3, 10, 30, and 60 μg/ml concentration for 72 h followed by lysis in cold RIPA buffer (50 mM Tris, pH 8.0, 150 mM NaCl, 1.0% NP-40, 0.5% sodium deoxycholate, 0.1% SDS, freshly supplemented with PhosSTOP (Roche, Germany) and complete protease inhibitor (A32963, Thermo Scientific, U.S.A.). The protein quantification of whole cell lysates was determined with Bradford Assay (G Bioscience, India). Equal protein for each sample prepared in 6X SDS loading Dye was resolved in 10% SDS-PAGE and subsequently transferred to 0.2 μm PVDF membrane (1620177, Bio-Rad, Hercules, CA, U.S.A.). Antibody dilutions, incubation time, and temperature conditions were according to the manufacturer’s protocol. Blots were developed using chemiluminescent HRP substrate (ECL, Merck-Millipore, U.S.A.) in Image Quant LAS 500 (GE Healthcare, U.S.A.) instrument for chemiluminescence detection. Immunoblots were further processed using Image Quant TL software provided with the instrument. Primary antibodies against phospho-ERK1/2 Thr 202, Tyr 204 (36-8800), ERK1/2 (13-6200), phospho-GSK3β Ser9 (MA5-14873), GAPDH (MA5-15738) were procured from Invitrogen, U.S.A. MITF (2535S) was purchased from Cell Signalling Technology (Danvers, MA, U.S.A.). All primary antibodies were used at 1:1000–1:2000 dilution. Anti-rabbit and anti-mouse HRP-tagged secondary antibodies (Invitrogen, U.S.A.) were used at a dilution of 1:10,000.

### Ultra-performance liquid chromatography/quadrupole time of flight mass spectrometry (UPLC/QToF-MS)

Melanogrit at 5 mg/ml concentration was prepared in methanol; sonicated, and filtered through 0.2 μm nylon filter. It was analyzed on Xevo G2-XS QToF with Acquity UPLC-I Class in the positive and negative mode of ionization with Unifi software (Waters Corporation, Milford, MA, U.S.A.). The mass spectrometry with electrospray ionization (ESI) was operated in MSE mode in a mass range of 50–1200 *m*/*z*. Other parameters include the acquisition time of 30 min, low and high collision energy set at 6 eV and 15–50 eV (ramp), respectively with a cone voltage of 40 V, capillary voltage of 1 kV for positive and 2 kV for negative mode. Source temperature of 120°C and desolvation temperature of 500°C, cone gas flow at 50 L/h, and desolvation gas flow at 900 L/h were other internal parameters optimized. Mass accuracy was maintained using 0.2 ng/ml of external reference (lock spray with leucine enkephalin) infused at a flow rate of 10 μl/min to generate a reference ion for the negative ion and positive ion mode at *m/z* 554.2620 and *m/z* 556.2766, respectively. The scan time for lock spray was assigned as 0.5 s with an interval of 30 s.

The separation of the analyte was carried out using the Acquity UPLC HSS T3 column (Waters Corporations, U.S.A., Part no.: 186003539) (100 × 2.1 mm, 1.8 µm). The column and analyte temperatures were maintained at 40 and 20°C, respectively, during the analysis. Approximately 1 µl of sample solution was injected into the column. The elution was carried out at a flow rate of 0.3 ml/min using gradient elution of mobile phase 0.1% formic acid in water (mobile phase A) and 0.1% formic acid in acetonitrile (mobile phase B). The volume ratio of mobile phase B varied as follows: 5–10% for 0–5 min, 10–15% for 5–15 min, 15–45% for 15–40 min, 45–55% for 40–55 min, 55–80% for 55–65 min, 80–5% for 65–66 min and 5% for 66–70 min. Compounds were analyzed by their respective mass-to-charge ratio (*m*/*z*) and fragmentation pattern. The *m*/*z* ratio was selected based on the molecular ions of these compounds [14]. Data acquisitions were collected under the positive and negative modes of ionization using full-spectrum scan analysis.

### Ultra-high-performance liquid chromatography (UHPLC) analysis

Protocatechuic acid, methyl gallate, vanillic acid, psoralene, and isopsoralene were dissolved independently in methanol to prepare the 1000 ppm of standard solution of each. A 50 ppm working standard mix solution was prepared by mixing 50 μl from each standard. Melanogrit in solution was prepared at 50 mg/ml concentration in methanol:water (80:20). The Melanogrit solution was sonicated and filtered through 0.45 μm nylon filter before subjecting to UHPLC (Shimadzu Prominence XR, Japan) armed with quaternary pump (Nexera XR LC-20AD XR) comprising degassing unit (DGU-20A 5R), DAD detector (SPD-M20 A) and Auto-sampler (Nexera XR SIL-20 AC XR) analysis. Chromatographic separation was achieved using a Shodex C18-4E (5 µm, 4.6*250 mm) column through binary gradient elution. Two solvents used for the analysis consisted of 0.05% glacial acetic acid in water (mobile phase A) and acetonitrile (mobile phase B). Gradient programming of mobile phase B was initially at 5–10% from 0 to 5 min, 5–15% from 5 to 10 min, 15–25% from 10 to 20 min, 25% from 20 to 30 min, 25–35% from 30 to 40 min, 35–50% from 40 to 50 min, 50–70% from 50 to 60 min, 70–90% from 60 to 65 min, 90–95% from 65 to 66 min, and 5% from 66 to 70 min with a flow rate of 1.0 ml/min. Approximately 10 µl of standard and test solution were injected and the column temperature was maintained at 35°C. The wavelength was set at 270 nm [15].

### Data analysis

GraphPad Prism 8.0 and MS office Excel 2010 was used to execute statistical calculations. Data sets of each group are expressed as mean ± standard error of mean (SEM) unless indicated. *P*-values for the data sets were considered significant if *P*<0.05 (*P<0.05, **P<0.005, ***P<0.0005, ****P<0.00001) and not significant (ns) if *P*>0.05. To determine the *P*-values, analysis of the mean values was performed through one- or two-way analysis of variance (ANOVA) with Tukey’s or Dunnett’s multiple comparison test.

## Results

### Melanogrit contains signature phytometabolites that are known to promote melanogenesis

**Figure 1:**
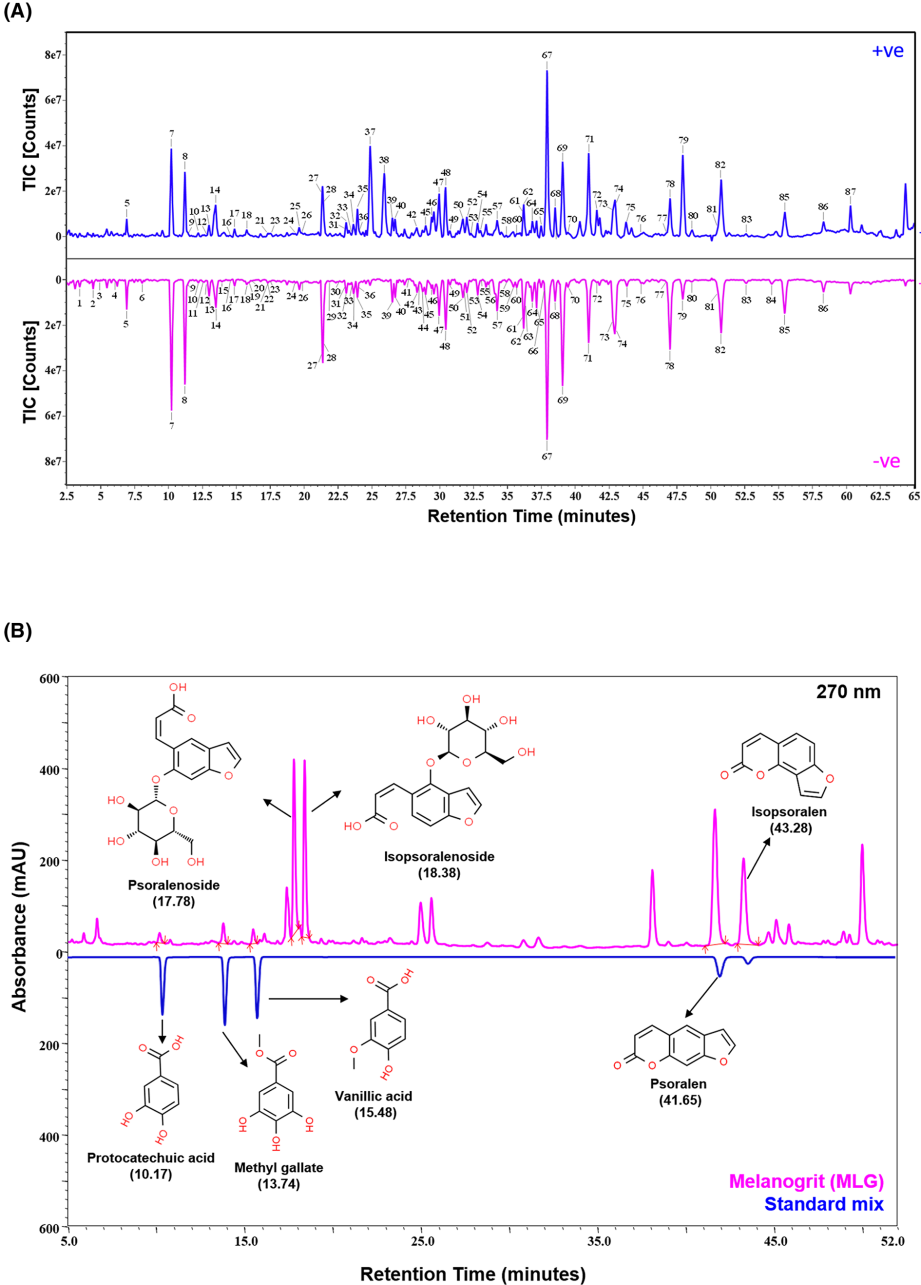
Melanogrit contains signature phytometabolites that are known to promote melanogenesis (**A**) The chromatograms of total ion count (TIC) versus retention time (minutes) depict ion acquisition in the positive (blue) and negative (pink) modes. Peaks in the chromatograms are represented with a number that can be referred in Table 1 for the phytometabolite identified by UPLC/QToF-MS in that peak. (**B**) Overlayed chromatogram generated through UHPLC showing the absorbance and retention time of standard mix (blue) and Melanogrit (pink). The peaks in Melanogrit aligned with the peaks of the standards with respect to their respective retention time are labeled with arrows and their chemical structures.

Melanogrit is composed of dry extracts prepared from seeds, leaves, roots, and whole plants of *Psoralea corylifolia, Acacia catechu, Rubia cordifolia*, and *Cassia fistula*, respectively (Table 1). Ayurveda, the meticulously structured Indian traditional medicinal system, has mentioned these plants with the ability to cure skin anomalies. Melanogrit was formulated as an herbal-mineral combination using approximately 87% of these plant extracts and 3.6% of mineral ingredients, specifically *Ras manikya* and *Tamra Bhasma* (Table 1). Melanogrit was characterized for the phytoconstituents through UPLC/MS-QToF and UHPLC approach. Total ion count (TIC) chromatogram generated through UPLC/MS-QToF identified a total of 87 phytoconstituents, through positive and negative mode (Figure 1A). Interestingly, the phytometabolites identified reflected the presence of all the plant components present in Melanogrit. The dominance of posralen and its corresponding analogues in the identified phytometabolites reflected the fact that *Psoralea corylifolia* is the main herbal component of Melanogrit, accounting for approximately 46% of all plants. *Acacia catechu* could have served as the major source of protocatechuic acid, caffeic acid, gossypin, kaempferol, baicalin, baicalein, ellagic acid, and chrysin. According to the previously published reports, roots of *Rubia cordifolia* probably were the source of munjistin, alizarin, purpurin and glycosides. Fruit of *Cassia fistula*, as reported earlier, plausibly sourced phytochemicals like, sinapic acid, glucosides, rhein, chrysophanein, and schaftoside. In addition, there were several other phytometabolites that were identified and listed in Table 2.

**Table 1:** Herbo-mineral composition of Melanogrit

Plant species/mineral	Plant part used	Family	Vernacular names		Weight/tablet (mg)
			Hindi	English	
*Psoralea corylifolia*	Fruit	Fabaceae	Bakuchi (Shodhit)	Purple fleabane	230
*Acacia catechu*	Bark	Fabaceae	Khadir	Cutch tree	55
*Rubia cordifolia*	Whole plant	Rubiaceae	Manjishtha	Indian madder	85
*Cassia fistula*	Fruit	Fabaceae	Amaltas	Indian laburnum	110
*Herbally processed mineral calyx*	–	–	Ras Manikya	–	10
*Herbally processed copper calyx*	–	–	Tamra Bhasma	–	10

**Table 2:** List of phytometabolites identified in Melanogrit through UPLC/QToF MS

S. No.#	Component name	Formula	Expected neutral mass (Da)	Observed mass (*m/z*)	Mass error (mDa)	RT (min)	Response	Adducts
1	Protocatechuic acid	C_7_H_6_O_4_	154.0266	153.0187	-0.6	3.45	49658	-H
2	1-O-Acetyl-6-deoxyhexopyranose	C_8_H_14_O_6_	206.0790	205.0711	-0.6	4.44	304252	-H
3	Caffeic acid 3-O-glucuronide	C_15_H_16_O_10_	356.0744	355.0666	-0.5	4.93	49822	-H
4	Caffeic Acid 4-β-D-Glucuronide	C_15_H_16_O_10_	356.0744	355.0668	-0.3	6.09	67301	-H
5	α-L-Glucopyranosyl 6-O- (2-methylbutanoyl)-α-L-glucopyranoside	C_17_H_30_O_12_	426.1737	449.1624 471.1718	-0.5 -0.2	6.91 6.92	257486 906489	+Na +HCOO
6	Gossypin	C_21_H_20_O_13_	480.0904	479.0829	-0.2	8.05	91081	-H
7	Psoralenoside	C_17_H_18_O_9_	366.0951	389.0838 365.0874	-0.5 -0.4	10.21 10.22	1099278 3431053	+Na -H
8	Isopsoralenoside	C_17_H_18_O_9_	366.0951	389.0838 365.0873	-0.5 -0.5	11.20 11.21	948631 2657846	+Na -H
9	Kaempferol-3-O-β-D-glucoside-7-O-α-L-arabinofuranoside	C_26_H_28_O_15_	580.1428	581.1506 579.1358	0.5 0.3	11.41 11.42	89424 112410	+H -H
10	Chrysophanein	C_21_H_20_O_9_	416.1107	417.1176 461.1091	-0.4 0.1	12.20 12.21	139683 122649	+H +HCOO
11	Sinapic acid	C_11_H_12_O_5_	224.0685	223.0608	-0.4	12.63	97066	-H
12	Schaftoside	C_26_H_28_O_14_	564.1479	565.1555 563.1407	0.3 0.1	12.87 12.87	88856 106026	+H -H
13	Cinncassiol A	C_20_H_30_O_7_	382.1992	383.2057 381.1915	-0.7 -0.4	13.00 13.00	156085 742071	+H -H
14	1,3-Dihydroxy-2-hydroxymethylanthraquinone-3-O-β-D -xylopyranose (1→6)-β-D-glucopyranoside	C_26_H_28_O_14_	564.1479	565.1552 563.1407	0.0 0.1	13.47 13.49	1466433 1989220	+H -H
15	3-Feruloylquinic acid	C_17_H_20_O_9_	368.1107	367.1032	-0.3	13.95	84040	-H
16	Lucidin primeveroside	C_26_H_28_O_14_	564.1479	565.1554 563.1407	0.2 0.1	14.55 14.56	46169 71995	+H -H
17	Isoschaftoside	C_26_H_28_O_14_	564.1479	565.1551 563.1410	-0.1 0.4	14.87 14.88	247360 378537	+H -H
18	Bavadin	C_27_H_30_O_13_	562.1686	585.1584 561.1614	0.5 0.1	15.76 15.77	80399 181858	+Na -H
19	4-Feruloylquinic acid	C_17_H_20_O_9_	368.1107	367.1031	-0.4	15.91	108471	-H
20	Ellagic acid	C_14_H_6_O_8_	302.0063	300.9987	-0.3	16.17	69581	-H
21	Isoquercetin	C_21_H_20_O_12_	464.0955	465.1021 463.0885	-0.7 0.3	17.29 17.30	17253 67121	+H -H
22	Baicalin	C_21_H_18_O_11_	446.0849	445.0779	0.3	17.33	72612	-H
23	Isocorylifonol	C_13_H_14_O_4_	234.0892	235.0955 233.0818	-1.0 -0.1	17.55 17.55	128639 38423	+H -H
24	Vicenin 1	C_26_H_28_O_14_	564.1479	587.1373 563.1408	0.1 0.2	19.36 19.37	28929 56598	+Na -H
25	Rubilactone	C_15_H_10_O_5_	270.0528	271.0589	-1.2	19.59	112391	+H
26	Astragalin	C_21_H_20_O_11_	448.1006	471.0894 447.0930	-0.4 -0.3	19.83 19.84	27100 110430	+Na -H
27	Vicenin 3	C_26_H_28_O_14_	564.1479	587.1377 563.1408	0.6 0.2	21.33 21.34	489649 1717608	+Na -H
28	Ruberythric acid	C_25_H_26_O_13_	534.1373	557.1271 533.1305	0.6 0.4	21.41 21.41	954403 2232897	+Na -H
29	Rubianic acid	C_25_H_26_O_13_	534.1373	533.1301	0.0	21.87	49285	-H
30	5,7-Dimethoxycoumarin	C_11_H_10_O_4_	206.0579	205.0501	-0.5	22.49	44826	-H
31	Coniferyl ferulate	C_20_H_20_O_6_	356.1260	357.1325 355.1187	-0.8 0.0	23.05 23.05	139919 116193	+H -H
32	Chrysin	C_15_H_10_O_4_	254.0579	255.0640 253.0505	-1.2 -0.1	23.12 23.12	567211 601144	+H -H
33	Irisflorentin	C_20_H_18_O_8_	386.1002	387.1070 385.0930	-0.5 0.1	23.44 23.44	54319 174599	+H -H
34	Pseudopurpurin	C_15_H_8_O_7_	300.0270	301.0334 299.0196	-0.9 -0.1	23.64 23.64	183816 332692	+H -H
35	Corylifol E	C_20_H_18_O_6_	354.1103	355.1168 353.1030	-0.8 -0.1	23.94 23.94	459678 729219	+H -H
36	Glycitein	C_16_H_12_O_5_	284.0685	285.0739 283.0604	-1.8 -0.8	24.10 24.10	59636 45112	+H -H
37	Psoralen	C_11_H_6_O_3_	186.0317	187.0379	-1.0	24.89	4364654	+H
38	Isopsoralen	C_11_H_6_O_3_	186.0317	187.0382	-0.8	25.90	3358061	+H
39	Rubiadinprimeveroside	C_26_H_28_O_13_	548.1530	571.1425 547.1459	0.3 0.2	26.52 26.52	322898 434385	+Na -H
40	Corylidin	C_20_H_16_O_7_	368.0896	369.0963 367.0821	-0.6 -0.3	26.72 26.72	337402 484900	+H -H
41	Baicalein	C_15_H_10_O_5_	270.0528	269.0453	-0.2	27.71	66452	-H
42	Coumestrol	C_15_H_8_O_5_	268.0372	269.0432 267.0296	-1.3 -0.3	28.36 28.36	131127 517676	+H -H
43	Rhein	C_15_H_8_O_6_	284.0321	283.0247	-0.1	28.53	95538	-H
44	Munjistin	C_15_H_8_O_6_	284.0321	283.0247	-0.1	28.78	678768	-H
45	Coryaurone A	C_20_H_18_O_6_	354.1103	355.1169 353.1029	-0.7 -0.2	28.97 28.97	387091 630340	+H -H
46	Bavachromanol	C_20_H_20_O_5_	340.1311	341.1376 339.1236	-0.8 -0.2	29.41 29.39	818710 444930	+H -H
47	Corylifol C	C_20_H_18_O_5_	338.1154	339.1220 337.1080	-0.7 -0.1	29.97 29.94	1118736 1683419	+H -H
48	Corylifol D	C_20_H_18_O_5_	338.1154	339.1221 337.1081	-0.6 0.0	30.42 30.42	1406877 1558851	+H -H
49	Brosimacutin F	C_20_H_20_O_6_	356.1260	357.1324 355.1184	-0.9 -0.3	30.78 30.78	138040 237600	+H -H
50	Alizarin	C_14_H_8_O_4_	240.0423	241.0485 239.0348	-1.0 -0.1	31.73 31.73	246082 678024	+H -H
51	Bavacoumestan A	C_20_H_16_O_6_	352.0947	351.0873	-0.1	31.79	162997	-H
52	Isopsoralidin	C_20_H_16_O_5_	336.0998	337.1062 335.0921	-0.8 -0.4	32.00 32.01	367958 547990	+H -H
53	5,4′-dihydroxy-6,7-furanbavachalcone	C_20_H_18_O_5_	338.1154	339.1217 337.1077	-1.0 -0.4	32.31 32.31	187827 154467	+H -H
54	Bakuchalcone	C_20_H_20_O_5_	340.1311	341.1376 339.1235	-0.7 -0.3	32.82 32.82	289691 620377	+H -H
55	Brosimacutin G	C_20_H_20_O_6_	356.1260	357.1329 355.1185	-0.4 -0.2	33.07 33.11	106204, 124826	+H -H
56	Bavacoumestan B	C_20_H_16_O_6_	352.0947	351.0872	-0.2	33.62	267114	-H
57	Psoralenol	C_20_H_18_O_5_	338.1154	339.1218 337.1080	-0.9 -0.1	34.24 34.24	663488 1414837	+H -H
58	Arctigenin	C_21_H_24_O_6_	372.1573	373.1635 371.1499	-1.0 -0.1	34.94 34.94	58747 168246	+H -H
59	Xanthopurpurin	C_14_H_8_O_4_	240.0423	239.0350	0.0	35.45	233611	-H
60	Purpurin	C_14_H_8_O_5_	256.0372	257.0432 255.0297	-1.3 -0.2	35.69 35.69	116931 477605	+H -H
61	Euchrenone a7	C_20_H_20_O_5_	340.1311	341.1374 339.1236	-1.0 -0.2	36.15 36.15	158083 254462	+H -H
62	Bavachalcone	C_20_H_20_O_4_	324.1362	325.1422 323.1287	-1.2 -0.2	36.21 36.20	1230268 2292972	+H -H
63	2′,4′-Dihydroxy-6′-methoxy-3′,5′-dimethylchalcone	C_18_H_18_O_4_	298.1205	297.1130	-0.2	36.29	308160	-H
64	Corylifol B	C_20_H_20_O_5_	340.1311	341.1373 339.1235	-1.0 -0.3	36.84 36.84	650069 1370577	+H -H
65	Euchrenone a2	C_25_H_26_O_5_	406.1780	429.1665 405.1707	-0.8 0.0	37.50 37.50	349555 414508	+Na -H
66	Ophioglonin	C_16_H_10_O_7_	314.0427	313.0353	0.0	37.79	227730	-H
67	Bavachromene	C_20_H_18_O_4_	322.1205	323.1269 321.1129	-0.9 -0.3	37.92 37.92	3404479 4561559	+H -H
68	Isobavachromene	C_20_H_18_O_4_	322.1205	323.1268 321.1132	-1.0 0.0	38.52 38.52	938093 1178414	+H -H
69	Corylifolinin	C_20_H_20_O_4_	324.1362	325.1423 323.1288	-1.1 -0.1	39.07 39.06	3084356 4286626	+H -H
70	Puerarone	C_20_H_16_O_5_	336.0998	337.1060 335.0925	-1.1 0.0	39.48 39.47	163452 305373	+H -H
71	Corylin	C_20_H_16_O_4_	320.1049	321.1115 319.0972	-0.6 -0.3	40.99 40.98	2729950 3179749	+H -H
72	Psorachromene	C_20_H_18_O_4_	322.1205	323.1270 321.1129	-0.8 -0.3	41.60 41.60	1026688 147231	+H -H
73	Isobavachalcone	C_20_H_20_O_4_	324.1362	325.1427 323.1286	-0.8 -0.3	42.80 42.79	1261512 2461672	+H -H
74	Psoralidin	C_20_H_16_O_5_	336.0998	337.1060 335.0921	-1.0 -0.4	42.95 42.95	1261517 3224449	+H -H
75	Psorachalcone A	C_20_H_20_O_5_	340.1311	341.1377 339.1236	-0.7 -0.2	43.80 43.79	74663 409607	+H -H
76	Bavachin	C_20_H_20_O_4_	324.1362	325.1425 323.1287	-0.9 -0.2	44.86 44.85	115557 257154	+H -H
77	Sophoracoumestan A	C_20_H_14_O_5_	334.0841	335.0905 333.0769	-0.9 0.1	46.74 46.73	208472 180879	+H -H
78	Isobavachin	C_20_H_20_O_4_	324.1362	325.1424 323.1289	-1.0 0.1	46.99 46.98	1692388 4623575	+H -H
79	Bavachinin	C_21_H_22_O_4_	338.1518	339.1583 337.1445	-0.8 0.0	47.94 47.93	4525476 804793	+H -H
80	1-Methoxyphaseollin	C_21_H_20_O_5_	352.1311	353.1375 351.1234	-0.8 -0.4	48.60 48.60	328001 131493	+H -H
81	Neobavaisoflavone	C_20_H_18_O_4_	322.1205	323.1268 321.1132	-0.9 0.0	50.57 50.56	499073 924998	+H -H
82	Corylifol A	C_25_H_26_O_4_	390.1831	391.1898 389.1757	-0.6 -0.1	50.76 50.76	2477880 4488431	+H -H
83	Isoneobavaisoflavone	C_20_H_18_O_4_	322.1205	323.1267 321.1131	-1.0 -0.2	52.61 52.60	118011 299819	+H -H
85	4-o-methylbavachalcone	C_21_H_22_O_4_	338.1518	339.1583 337.1443	-0.8 -0.2	55.45 55.45	1652412 3228741	+H -H
86	Neocorylin	C_25_H_24_O_4_	388.1675	411.1560 387.1600	-0.7 -0.2	58.32 58.33	381277 581877	+Na -H
87	Bakuchiol	C_18_H_24_O	256.1827	257.1890	-1.0	60.32	218835	+H

# represents the peak number depicted in the chromatogram (Figure 1A).

Furthermore, through UHPLC, we quantified and validated the presence of signature phytometabolites that were identified through UPLC/MS-QToF. Melanogrit and a standard mix of phytometabolites containing protocatechuic acid, methyl gallate, vanillic acid, psoralen, and isopsoralen were subjected to UHPLC. The retention time (RT) of indicated phytometabolites in the standard mix was matched to the chromatogram of Melanogrit generated at 250 nm to validate their respective presence (Figure 1B). Quantification further showed psoralen (7.227 μg/mg) as one of the major phytometabolite, while protocatechuic acid, methyl gallate, and vanillic acid were quantified at 0.175, 0.309, and 0.222 μg/mg, respectively (Table 3).

**Table 3:** UHPLC-based identification and quantification of phytochemicals present in Melanogrit

S.No.	RT	Phytochemical identified	Molecular weight (g/mol)	Content (μg/mg)
1	10.17	Protocatechuic acid	154.12	0.175
2	13.74	Methyl Gallate	184.15	0.309
3	15.78	Vanillic acid	168.14	0.222
4	17.78	Psoralenoside	186.16	9.503
5	18.38	Isopsoralenoside	186.16	9.442
6	41.65	Psoralen	186.16	7.227
7	43.28	Isopsoralen	186.16	11.711

### Melanogrit promotes dendrite formation in melanocytes

**Figure 2:**
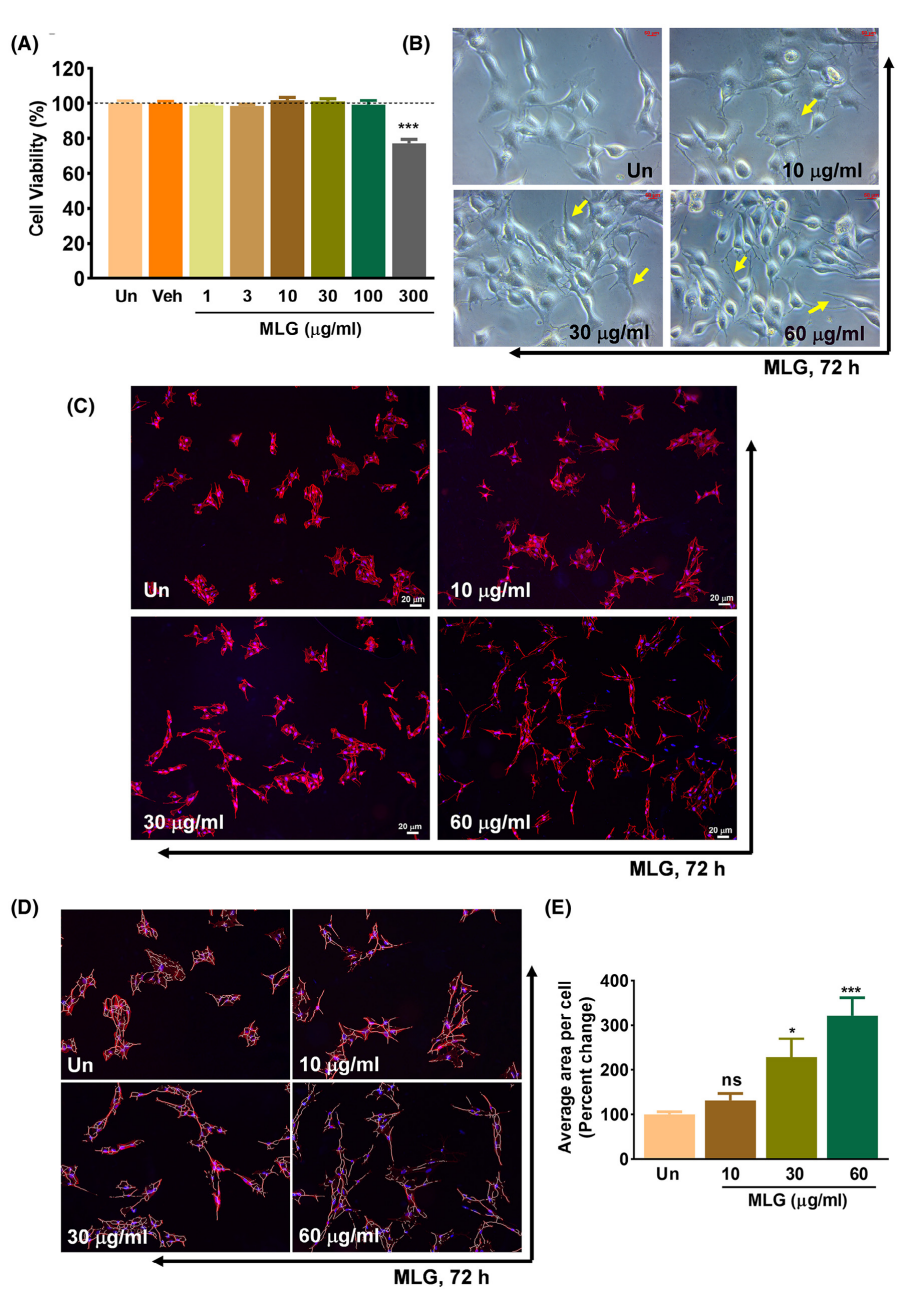
Melanogrit promotes dendrite formation in melanocytes (**A**) Percent cell viability in B16F10 cells treated with 1, 3, 10, 30, 100, and 300 μg/ml concentration of Melanogrit (MLG) for 72 h (*n*=3) was assessed through alamar blue assay. Percent cell viability was determined with respect to untreated (Un) cells. The vehicle indicated as ‘Veh’ in the bar graph is 0.9% DMSO present in 300 μg/ml of Melanogrit. A significant reduction in cell viability was evaluated in B16F10 cells when treated with 300 μg/ml of Melanogrit. Two-way ANOVA employing Tukey’s multiple comparison test (α = 0.05) was used to analyze significant differences in the cell viability of treated cells compared with untreated (Un). Error bars represent mean ± SEM; the significance of data is represented as, ***P<0.0005 and not significant (ns) if *P*>0.05. (**B**) B16F10 cells treated with 10, 30 and 60 μg/ml concentration of Melanogrit for 72 h. Cells were observed and captured under the brightfield microscope. (**C**) Alexa Fluor 594-Phalloidin stained B16F10 cells treated with Melanogrit at 10, 30 and 60 μg/ml concentrations. (**D**). Digitally zoomed images of panel (C) overlayed with the ImageJ generated automated tracings. These tracings were used by the software to determine the total area covered by the cells. (**E**) Graph illustrates the average area percentage change calculated with respect to untreated. Total area covered is normalized with the number of total cell count. Four images from each treatment in (C) were used for analysis. Two-way ANOVA employing Tukey’s multiple comparison test (α = 0.05) was used to analyze significant differences in the cell viability of treated cells compared with untreated (Un). Error bars represent mean ± SEM; the significance of data is represented as *P<0.05, ***P<0.0005 and not significant (ns) if P>0.05. MLG, Melanogrit.

B16F10 cells were treated with Melanogrit at concentrations ranging from 1 to 300 μg/ml for 72 h to determine the cytosafety of Melanogrit. The Alamar blue cell viability assay assessed that Melanogrit does not induce cytotoxicity up to 100 μg/ml concentration; however, at 300 μg/ml, the cell viability has reduced to 77% (Figure 2A). We, therefore, performed experiments at doses <100 μg/ml of Melanogrit. The treated cells showed a change in cellular morphology compared with the untreated. It is reported previously that the formation of dendrite extensions in melanocytes indicates the dispersion and transfer of melanosomes to the neighbouring keratinocytes. We observed under the bright field microscope that with the Melanogrit treatment, B16F10 cells became enlarged with elongated dendritic protrusions (Figure 2B). B16F10 cells were stained with Phalloidin-Alexa fluor 594 (Red) to capture the changes in the cytoskeleton. With the staining, it was determined that Melanogrit treatment resulted in an increase in cell size, and dose-dependently reinforced the length and number of dendrite extensions compared with the untreated B16F10 cells (Figure 2C). The ImageJ-based automated analysis produced tracings representing the area covered by the cells, and these tracings were then superimposed on to the original images for visual verification (Figure 2D). The total area encompassed by these tracings was normalized relative to the total cell count and depicted as a percentage change compared with the untreated condition. Melanogrit at concentration, 30 and 60 μg/ml significantly increased the percent area covered by the cells compared with untreated condition (Figure 2E). Taken together, Melanogrit-induced morphological changes are indicative of melanogenesis in B16F10 cells.

### Melanogrit up-regulates the decisive genes of melanogenesis pathway

**Figure 3:**
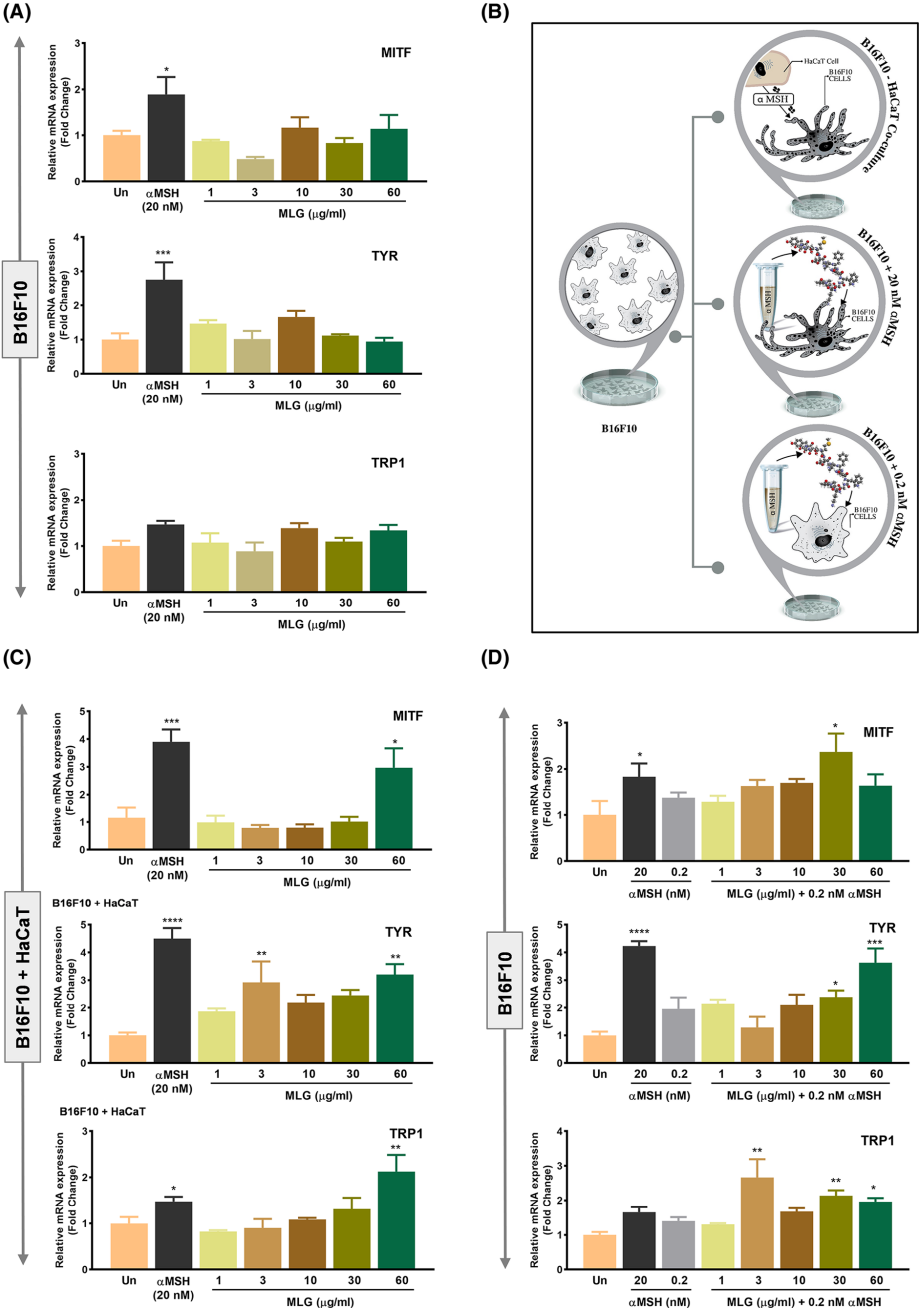
Melanogrit up-regulates the decisive genes of melanogenesis pathway (**A**) Relative gene expression levels of MITF, TYR, and TRP1 with respect to β-actin were determined in B16F10 cells treated with either 20 nM αMSH or indicated concentrations of Melanogrit for 72 h. (**B**) A schematic showing the three independent models of B16F10 cells we established to study melanogenesis. B16F10 cells when co-cultured with HaCaT cells show melanogenesis induction, 20 nM αMSH treatment in B16F10 cells also induces melanogenesis, whereas, a dose of 0.2 nM αMSH in B16F10 cells was unable to induce melanogenesis. (**C**) Relative gene expression levels of MITF, TYR, and TRP1 with respect to β-actin were determined in a co-culture of B16F10-HaCat cells treated with either 20 nM αMSH or indicated concentrations of Melanogrit for 72 h. (**D**) Relative gene expression levels of MITF, TYR, and TRP1 with respect to β-actin were determined in B16F10 cells treated with either 20 nM αMSH or indicated concentrations of Melanogrit for 72 h in the presence a of sub-optimal dose of αMSH (0.2 nM). Two-way ANOVA employing Tukey’s multiple comparison test (α = 0.05) was used to analyze significant differences in the indicated gene expression level of treated cells compared with untreated (Un). Error bars represent mean ± SEM; the significance of data is represented as **P<0.005, ***P<0.0005, ****P<0.0001, and not significant (ns) if *P*>0.05; MLG, Melanogrit. Symbol P indicate significance, *P<0.05. the significance of data is represented as *P<0.05, **P<0.005, ***P<0.0005, ****P<0.0001, and not significant (ns) if P>0.05.

The αMSH has already been reported to stimulate melanocytes for melanin production by up-regulating genes of the melanogenesis pathway. We, therefore, treated B16F10 cells with an optimal dose of αMSH (20 nM) that could serve as a positive control for the experiment [15]. The 20 nM αMSH treatment significantly up-regulated MITF and TYR; however, no significant change in gene expression levels of TRP-1 was observed. Melanogrit treatment, contrary to our expectations, showed no significant change in MITF, TYR, and TRP-1, irrespective of the concentration (Figure 3A). We next sought to develop an *in vitro* model wherein the inducer of melanogenesis could be tested. Previous research reports have shown that paracrine stimulation from keratinocytes induces melanin biosynthesis inside the melanocytes. We, therefore, cultured B16F10 melanocyte with HaCaT cells in the same well to address the importance of physical contact and paracrine stimulation from keratinocytes by the melanocytes. Another model is to trigger B16F10 cells with a sub-optimal dose of αMSH, which itself does not induce melanogenesis but could trigger basal paracrine signalling necessary for melanogenesis induction (Figure 3B). To investigate this, the gene levels of MITF, TYR, and TRP-1 were assessed in melanocytes B16F10 cells that were co-cultured with keratinocytes, HaCaT cells (Figure 3C). Interestingly, αMSH (20 nM) treatment in co-culture set up showed significantly higher gene expression levels of MITF, TYR, and TRP-1 compared with B16F10 alone in Figure 3A. Melanogrit at 60 μg/ml concentration showed a significant increase in MITF and TYR gene levels, and Melanogrit-mediated dose-dependent increase was observed in TRP-1 gene levels. Interestingly, B16F10 cells spiked with the sub-optimal dose of αMSH (0.2 nM), when treated with Melanogrit showed a significant up-regulation in all three genes. Of note, 0.2 nM αMSH treatment alone resulted in no significant increase in MITF, TYR, and TRP-1 gene levels (Figure 3D). Collectively, Melanogrit may potentiate melanogenesis at the gene level, however, an appropriate model is a prerequisite to determine these changes. We demonstrate either keratinocytes-melanocyte co-culture or sub-optimal αMSH-stimulated B16F10 are apt *in vitro* models to study the mechanism of melanogenesis inducers. This result, hence, illustrates that Melanogrit may be further investigated as a melanogenesis stimulator and a potential therapeutic treatment against vitiligo.

### Melanogrit escalates the cellular tyrosinase activity in melanocytes

**Figure 4:**
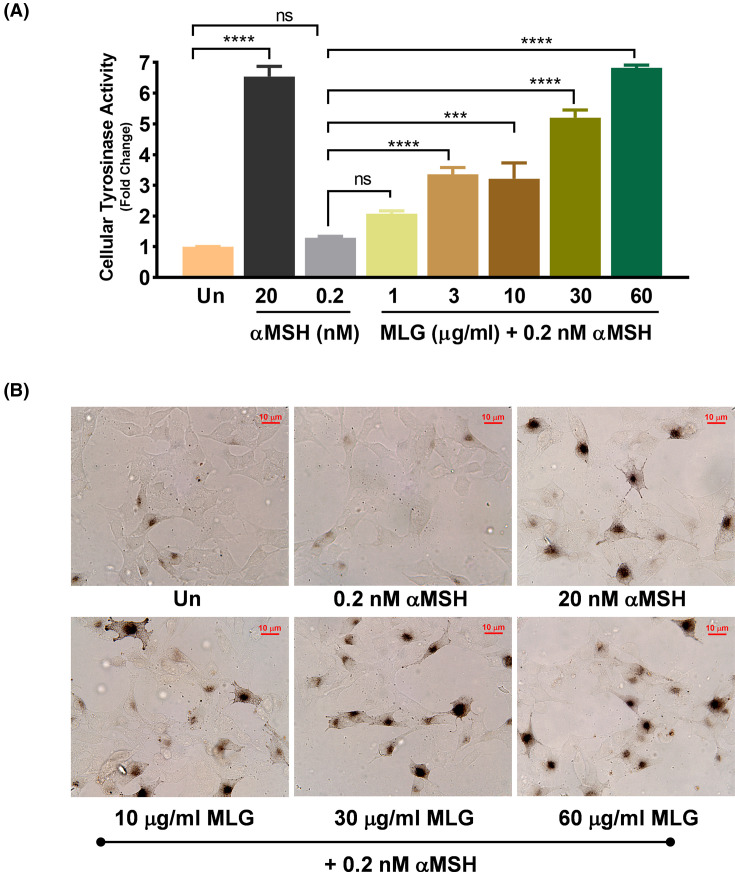
Melanogrit escalates the cellular tyrosinase activity in melanocytes (**A**) Relative fold change in the cellular tyrosinase activity with respect to the untreated (Un) was evaluated in the whole cell lysates of B16F10 cells treated with 20 nM αMSH or indicated concentrations of Melanogrit for 72 h in the presence a of sub-optimal dose of αMSH (0.2 nM). Two-way ANOVA employing Tukey’s multiple comparison tests (α = 0.05) was used to analyze significant differences in the indicated gene expression level of treated cells compared with untreated (Un). Error bars represent mean ± SEM; the significance of data is represented as **P<0.005, ***P<0.0005, ****P<0.0001, and not significant (ns) if *P*>0.05. (**B**) Brightfield images of B16F10 cells either treated with indicated doses of Melanogrit or αMSH showing L-DOPA staining. The black coloration in cells indicates higher staining of L-DOPA due to higher cellular tyrosinase activity; MLG, Melanogrit. Two-way ANOVA employing Tukey’s multiple comparison tests (α = 0.05) was used to analyze significant differences in the indicated gene expression level of treated cells compared with untreated (Un). Error bars represent mean ± SEM; the significance of data is represented as ***P<0.0005, ****P<0.00001, and not significant (ns) if *P*>0.05; MLG, Melanogrit.

The copper-containing enzyme tyrosinase (TYR) activity is a prerequisite for melanin biosynthesis. It is located in the membrane of the melanosomes wherein it regulates the rate-limiting step of converting tyrosine or L-DOPA into eumelanin or pheomelanin [2,16]. With Melanogrit treatment, we witnessed a significant rise in the TYR gene expression levels, albeit it was also critically important to assess the intracellular tyrosinase enzyme activity. L-DOPA, the reaction substrate was incubated with the whole cell lysates of Melanogrit treated B16F10, as indicated. The whole cell lysates facilitate the tyrosinase enzyme that subsequently catalyses the oxidation of L-DOPA into dopaquinone. The rate of L-DOPA oxidation can be assessed by monitoring the absorbance of the reaction at 475 nm. Treatment of 20 nM αMSH showed an increase of tyrosinase activity of more than 6-fold compared with untreated. A combination of Melanogrit and 0.2 nM αMSH treatment also showed an increased cellular tyrosinase activity by more than 6-fold, in a dose-dependent manner, compared with 0.2 nM αMSH alone (Figure 4A). L-DOPA staining was then directly performed in B16F10 cells treated with different concentrations of Melanogrit. Cells turning black in the presence of L-DOPA indicate heightened cellular tyrosinase activity. Under the brightfield microscope, it was observed that cells treated with the combination of Melanogrit and 0.2 nM αMSH, dose-dependently enhanced the black color in the cells. However, 0.2 nM αMSH alone was corresponding to the untreated B16F10 cells whereas 20 nM αMSH showed cells turning black in the presence of L-DOPA (Figure 4B). Altogether, the results indicate that Melanogrit potentiates melanogenesis by escalating the cellular tyrosinase activity.

### Melanogrit substantially boosted melanin production in melanocytes

**Figure 5:**
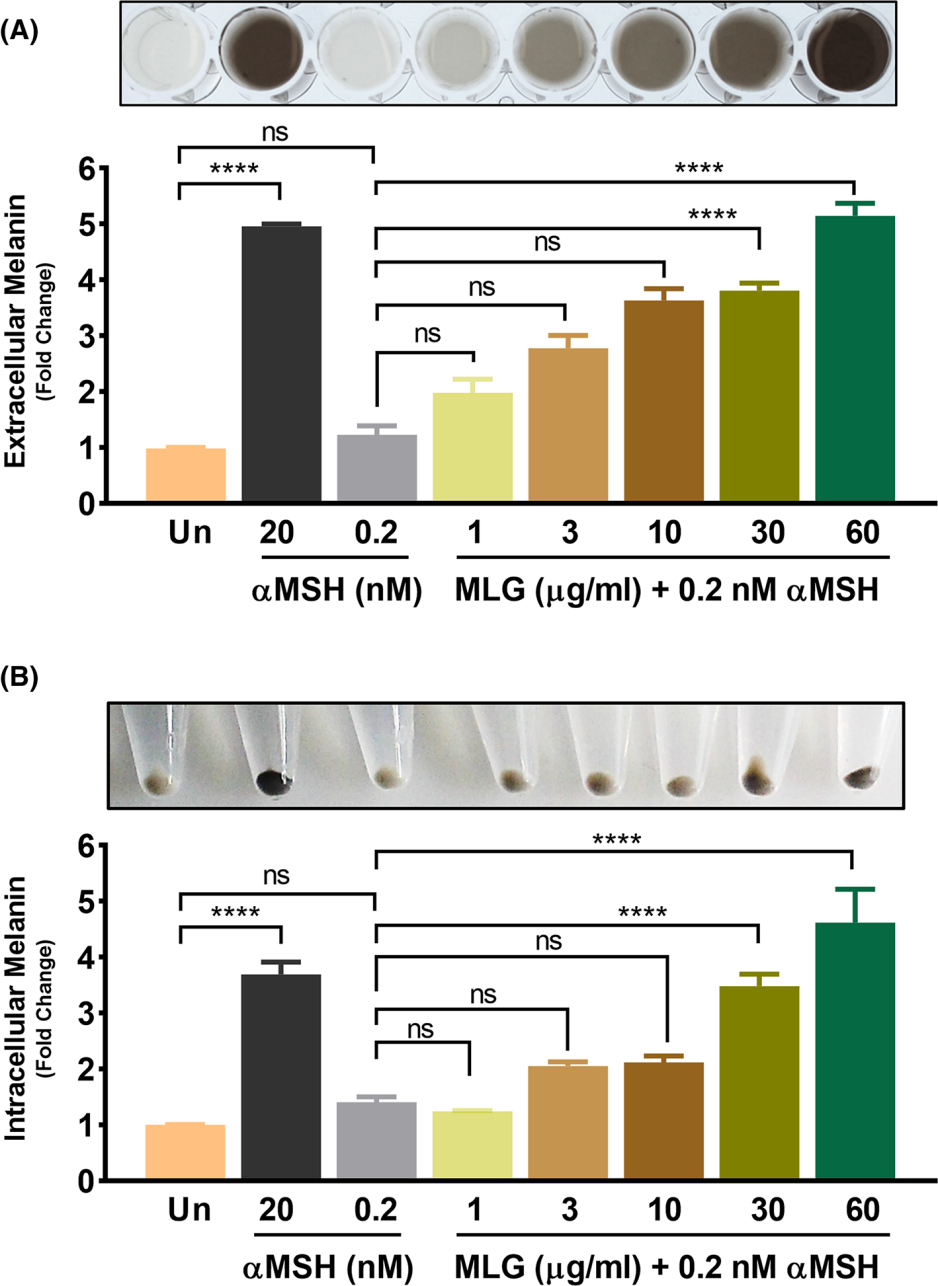
Melanogrit substantially boosted melanin production in melanocytes (**A**) The upper panel is the image of the supernatant of B16F10 cells treated with 20 nM αMSH or indicated concentrations of Melanogrit for 72 h in the presence a of sub-optimal dose of αMSH (0.2 nM). Lower panel is the melanin quantification in the respective media supernatant collected. (**B**) Upper panel is the image of the cell pellet of B16F10 cells treated with 20 nM αMSH or indicated concentrations of Melanogrit for 72 h in the presence a of sub-optimal dose of αMSH (0.2 nM). Lower panel is the melanin quantification of the respective cell pellet. Two-way ANOVA employing Tukey’s multiple comparison tests (α = 0.05) was used to analyze significant differences in the indicated gene expression level of treated cells compared with untreated (Un). Error bars represent mean ± SEM; the significance of data is represented as ****P<0.00001, and not significant (ns) if *P*>0.05; MLG, Melanogrit.

A treatment of αMSH at 20 nM turned the phenol red-free, colorless media of B16F10 cells into black, sign of melanin production by the cells. The above optimized B16F10 model that was sub-optimally stimulated with Melanogrit 0.2 nM αMSH was employed for determining the impact of Melnoagrit on melanin production. The sub-optimal dose of αMSH, i.e., 0.2 nM displayed no color change and was comparable to untreated. However, Melanogrit treatment in the same setting, concomitantly increased the black color intensity of the colourless media, indicative of melanin production. It is the extracellular melanin that is secreted out of the melanocytes that turn the media into black in color (Figure 5A, upper panel). To quantify the extracellular melanin, the color was measured against synthetic melanin standard and converted into fold change with respect to the untreated. Treatment of a higher concentration (20 nM) of αMSH served as positive control for the experiment. Notably, Melanogrit at 60 μg/ml concentration and αMSH at 20 nM, both showed ∼5-fold increase in extracellular melanin production (Figure 5A, lower panel). A qualitative and quantitative dose-dependent effect was indeed observed with Melanogrit treatment. We then quantitated the intracellular melanin levels in the B16F10 cells treated with αMSH (20 nM, 0.2 nM) and different doses of Melanogrit. The cell pellets collected showed cells turning black under specific treatment conditions (Figure 5B, upper panel) compared with the untreated or 0.2 nM αMSH. The quantified intracellular melanin coincided with the black color intensity of the respective cell pellet. Intracellular melanin levels increased to approximately 4-, 4.5- and 3.5-fold with 20 nM αMSH, 30 μg/ml and 60 μg/ml of Melanogrit, respectively (Figure 5B, lower panel). However, 0.2 nM αMSH alone or Melanogrit at 1, 3, and 10 μg/ml showed non-significant (ns) changes in the intracellular melanin levels.

### Melanogrit up-regulates MITF by inhibiting ERK activation

**Figure 6:**
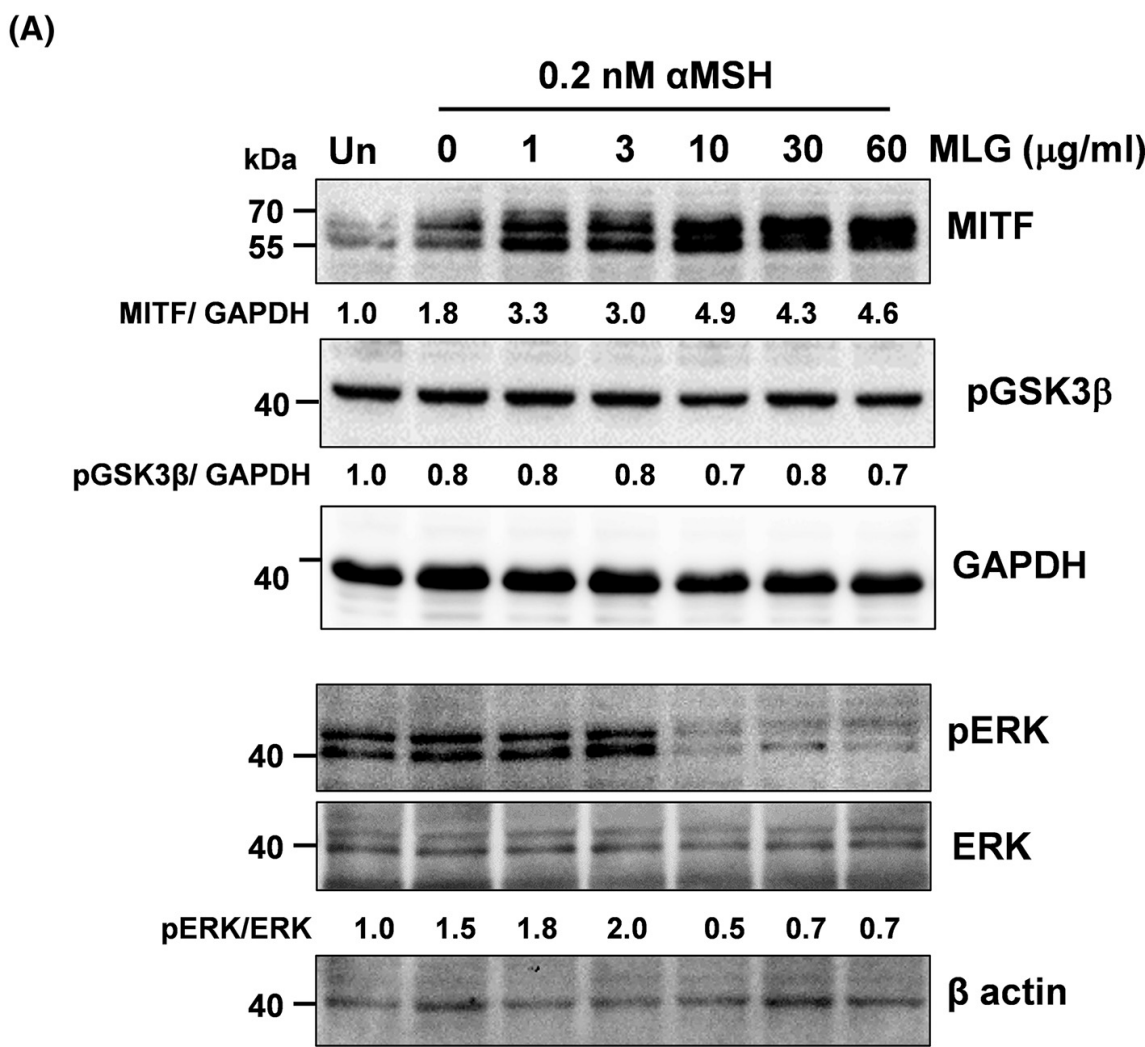
Melanogrit up-regulates MITF by inhibiting ERK activation (**A**) Whole cell lysates of B16F10 cells post 72 h of indicated doses of Melanogrit and αMSH treatment were subjected to Western blotting. Immunoblots were developed using antibodies against MITF, pERK, pGSK3β, ERK, and GAPDH, β-actin; MLG, Melanogrit.

In order to thoroughly comprehend the mechanism of action of Melanogrit, it was crucial to ascertain the translated levels of the key transcription factor, MITF. We determined the MITF protein levels in the whole cell lysates of B16F10 cells treated with a combination of Melanogrit and 0.2 nM αMSH for 72 h. MITF protein levels showed a dose-dependent gradual increase with Melanogrit treatment and therefore, assessed the levels of pGSK3β and pERK levels, the kinases that have previously been shown to regulate the proteasomal degradation of MITF. Interestingly, pERK levels diminished at 10, 30, and 60 μg/ml of Melanogrit treatment; however, the levels of pGSK3β were observed to be almost similar with Melanogrit treatment (Figure 6). pERK levels normalized with whole ERK showed significant down-regulation, whereas, MITF expression levels normalized with GAPDH showed significant up-regulation.

## Discussion

The healing potential of plants against various skin abnormalities has been widely studied. Several plants like *Daphne gnidium, Moricandia arvensis, Cassia alata, Cassia occidentalis, Polygonum multiflorum, Eclipta Prostrate, Pyrostegia venusta, Vernonia anthelmintica, Melissa officinalis, Melia azedarach, Capparis spinosa, Erica multiflora, Citrus paradisi, Citrus grandis*, and *Fructus aurantia* have been studied for their potential as stimulants of melanogenesis through various *in vitro* and *in vivo* experiments [17,18]. The herbal composition of Melanogrit is composed of four plants, *Psoralea corylifolia, Acacia catechu*, *Rubia cordifolia*, *Cassia fistula*, all well known for their effective medicinal qualities for alleviating a variety of illnesses, including those related to the skin (Table 1). Individually, *Acacia catechu* is recommended in medicinal preparations for the treatment of gastro-intestinal ailments, asthma, dental infections, leprosy, leukoderma, and skin diseases. Research studies have also reported the antioxidant, antimicrobial, and immunomodulatory properties in the leaves and heartwood of *Acacia catechu* [19]. Pharmacological properties of the different plant parts of *Cassia fistula*, involving fruit, flower, leaves, bark, and seeds have been shown to be effective against skin diseases, liver, abdomen, gland, throat, and lung ailments [20]. *Rubia cordifolia* in the list is traditionally used for treating skin disorders. Additionally, research studies have also unravelled the hepatoprotective, anti-cancer, anti-microbial immunomodulatory, and anti-oxidant properties of *Rubia cordifolia* through various *in vitro* and *in vivo* models [21,22]. *Psoralea corylifolia*, the major component of Melanogrit not only holds a notable place in the Traditional Indian Medicine system but also in Traditional Chinese Medicine (TCM), wherein it is prescribed for the treatment of spermatorrhoea, osteoporosis, leprosy, and various skin disorders. The herbal extracts prepared from the seeds of *Psoralea corylifolia* demonstrate pharmacological activities, that include anticancer, antimicrobial, anti-inflammatory, antioxidative, and neuroprotective [23–25]. The seeds of *Psoralea corylifolia* make up to 42% of Melanogrit, also readily apparent from the phytochemical profile. We obtained a wide range of flavonoids from *Psoralea corylifolia*, including, psoralenoside, isopsoralenoside, psoralen, isopsoralen, isopsoralidin, psoralenol, 5,4′-dihydroxy-6,7-furanbavachalcone, bakuchalcone, psoralen, isopsoralen, corylidin, corylifol C, corylifol D, isopsoralidin, 5,4′-dihydroxy-6,7-furanbavachalcone, bakuchalcone, psoralenol, bavachalcone, 2′,4′-dihydroxy-6′-methoxy-3′,5′-dimethylchalcone, corylifol B, corylifolinin, corylin, isobavachalcone, psoralidin, bavachin, isobavachin, bavachinin, corylifol, A3-hydroxybakuchiol, 4-O-methylbavachalcone, bakuchiol; few of them have even been studies scientifically for their utilization in the treatment of vitiligo [25]. Notably, Kaempferol identified in our study has earlier been reported as a melanogenesis stimulator [26]. Hence, the combination of herbs has made Melanogrit a diversified source of phytochemicals (Figure 1 and Table 2).

Current treatments for vitiligo involve the application of topical corticosteroids and calcineurin inhibitors to regulate the local immune response and stimulate melanocytes for pigment production. Topical vitamin D analogues and antioxidants have also been shown to yield moderate results in treating vitiligo patients. Interestingly, the topical treatment modalities are at times combined with therapies like excimer laser or narrowband UVB to enhance the treatment benefits. The mainstay of treatment for unstable vitiligo has been topical agents and phototherapy. However, systemic treatments are increasingly being shown to have a significant impact on the course of the disease as monotherapy or adjunctive therapy [27]. Of note, oral mini‐pulsed corticosteroid therapy, methotrexate, minocycline, cyclosporin, Janus kinase inhibitors and certain supplements have been used in the systemic treatment of vitiligo. Janus kinase (JAK) inhibitors such as tofacitinib and ruxolitinib exhibit potential by modulating immune responses. Monoclonal antibodies targeting various molecules, including smaller proteins like affibodies and nanobodies, are the promising line of treatment for vitiligo. Furthermore, the Wnt/β-catenin signaling pathway plays a pivotal role in melanocyte proliferation, considered as potential adjunct therapy for vitiligo [27–29]. Our study on dissecting the mechanism of action of Melanogrit determined it to largely act as an ERK modulator that influences MITF levels, a key regulator of melanogenesis, independent of GSK3β. It is associated with ERK activation, which subsequently up-regulates MITF levels, offering a potential mechanism that could be targeted in the treatment of vitiligo. Indeed, this is consistent with previous findings that have demonstrated ERK as a regulator of MITF levels [30–32].

The initial findings of our study determined Melanogrit to be cytosafe even at a high dose of 100 μg/ml; furthermore, Melanogrit induced morphological changes in the melanocytes that are indicative of melanogenesis (Figure 2). Melanocytes showed an increase in cell size and dendritic extensions, which are thought to be essential for the active transfer of melanin from melanocytes to keratinocytes [33,34]. The bright field images and automated quantification of phalloidin staining showed numerous dendritic protrusions stretching out of B16F10 cells with Melanogrit treatment (Figure 2B–E). The results indicated at the active transport of melanosomes with Melanogrit treatment that could potentially increase the melanin production through the melanocytes in the vicinity of depigmented areas. The increased dendricity and enhanced melanogenesis, both mechanisms could subsequently lead to skin repigmentation. However, the potential of Melanogrit in retrieving the lost melanocyte in the white skin patches needs to be explored further.

Contrary to our anticipation, Melanogrit by itself could not induce the transcription levels of hallmark genes, namely, MITF, TYR, and TRP1, which regulate melanogenesis or biosynthesis of melanin. Although, numerous reports have used B16F10 cells as *in vitro* model system to test melanogenesis inhibitors and stimulators, but we hypothesized that perhaps a physical contact or presence of keratinocytes in the vicinity was obligatory for melanocytes to trigger melanogenesis [34,35]. We co-cultured B16F10 cells from murine lineage and HaCaT cells from human lineage in the same well, thereby allowing both the melanocytes and keratinocytes to establish physical interaction and associated signalling [36,37]. We were keen to determine the gene levels of MITF, TYR, and TRP1 in the co-cultured B16F10 cells (Figure 3). However, keratinocytes of the murine lineage in the co-culture could make it challenging to determine MITF, TYR, and TRP1 levels only in B16F10 cells. Therefore, we purposely employed the HaCaT cell line of the human lineage. The primers, indeed used for MITF, TYR, and TRP1 levels were checked for any unspecific amplification in HaCaT cells before, establishing the experiment. The results in Figure 3C showed a robust increase of MITF, TYR, and TRP1 in the co-cultured B16F10 cells when treated with Melanogrit, albeit only at a concentration of 60 μg/ml. We further addressed the necessity of the physical contact between the melanocyte and keratinocytes in our system, by simply treating B16F10 cells with sub-optimal dose of αMSH, wherein it could only mimic as a paracrine signal for melanocytes secreted through keratinocytes. Surprisingly, this model of Melanogrit showed a significant increase in MITF, TYR, and TRP1 gene expression levels. αMSH is known to promote the production of melanin and potentially leading to repigmentation of depigmented skin areas. αMSH acts as a paracrine hormone on melanocytes by binding to their surface MC1R receptors, inducing the expression of enzymes required for the synthesis of melanin and photoprotective eumelanin [38]. Studies utilizing a synthetic analog of αMSH, afamelanotide have shown, it stimulates melanogenesis and addresses the inflammatory microenvironment in vitiligo lesions. Clinical trials have investigated its efficacy and safety in vitiligo. A combination of afamelanotide and narrowband UVB phototherapy (NB-UVB) outperformed phototherapy alone, resulting in significant repigmentation [39,40]. Considering the importance of αMSH in the melanogenesis pathway, we used a sub-optimal dose of αMSH to mimic physiologically relevant conditions under *in vitro* settings. The suboptimal utilization of αMSH likely prompted melanocytes to trigger melanogenesis pathway that had not been previously observed with Melanogrit treatment alone (as depicted in Figure 3A). It is noteworthy that if we had employed a higher dose of αMSH, it might have obscured the therapeutic potential of Melanogrit, probably because both Melanogrit and αMSH activate the melanogenesis pathway through similarroute. This approach mimics the availability of αMSH to melanocytes in a manner similar to the physiological condition, where melanocytes receive αMSH from neighbouring keratinocytes [41]. We determined that Melanogrit not only increased the transcription levels of the copper-containing enzyme tyrosinase (TYR) but also helped escalate its intracellular activity. We further validated the effect of Melanogrit on tyrosinase activity with L-DOPA staining of the cells (Figure 4). The tyrosinase enzyme inside the cells react with L-DOPA present extracellularly. This converts the cells with high cellular tyrosinase activity to black. Melanogrit treatment increased the number of black cells and their respective intensity with L-DOPA staining. The varied phytometabolic profile of Melanogrit may be accountable for modulating the tyrosinase activity. This has opened new direction for our research and has prompted us to investigate how phytochemicals can increase tyrosinase activity. In addition, a robust increase in the extracellular and intracellular melanin levels in response to Melanogrit treatment first, validated the potential of Melanogrit as melanogenesis stimulator and secondly, the study model, wherein 0.2 nM αMSH had no significant changes with untreated (Figure 5). To further unravel the mechanism, we determined the direct or indirect impact of Melanogrit on protein levels of MITF. Melanogrit increased the protein expression of MITF while also strongly inhibiting the levels of ERK activation. The inverse expression levels of pERK and MITF, suggested an indirect regulation of MITF by Melanogrit via pERK (Figure 6). Previously, research studies have shown that MITF undergoes phosphorylation at Ser 73 and Ser403 by kinases, ERK and GSK3β that promote ubiquitin proteasomal degradation of MITF; however, the two events are independent [42,43]. We, therefore, assessed the expression levels of pGSK3β and observed no significant alteration with Melanogrit, indicating ERK-mediated regulation of MITF with Melanogrit.

**Figure 7:**
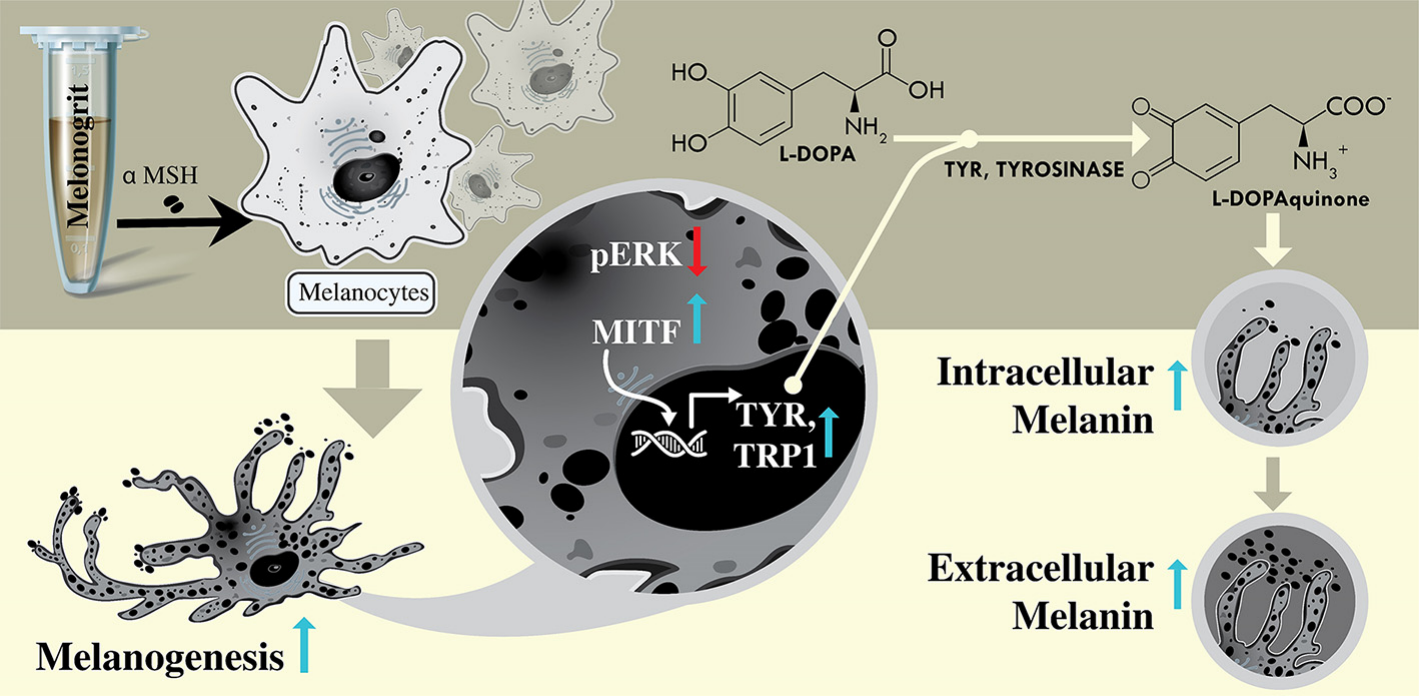
Proposed summarization model The diagrammatic illustration shows that 0.2 nM αMSH induction in B16F10 cells does not manifest any measurable effects, however, when combined with Melanogrit triggered melanogenesis. Melanogrit transcriptionally upregulated the melanogenesis pathway via MITF, TYR, and TRP1; which in turn escalated cellular tyrosine activity and melanin biosynthesis. Melanogrit ameliorated MITF protein levels by inhibiting pERK.

Taken together, the polyherbo-mineral formulation, Melanogrit exhibits strong potential to induce melanogenesis pathway. It could modulate the melanogenesis pathway at transcript, translation, and even post-translational levels as well (Figure 7). This formulation could further be evaluated either alone or in combination with phototherapy for the development of anti-vitiligo therapy with better efficacy and cytosafety.

## Data Availability

The data that support the findings of this study are available from the corresponding author upon reasonable request.

## Abbreviations

αMSH: α-melanocyte stimulating hormone
ACTH: adrenocorticotropic hormones
DMSO: dimethyl sulphoxide
ET-1: endothelin-1
JAK: Janus kinase
MLG: Melanogrit
SCF: stem cell factor
TCM: Traditional Chinese Medicine
TIC: total ion count
TYR: tyrosinase
